# Analysis of Hydrothermal Systems Beneath Tayukeng through Long-Term Geochemical Signals of Hydrothermal Fluids in Tatun Volcano Group, Taiwan

**DOI:** 10.3390/ijerph18147411

**Published:** 2021-07-11

**Authors:** Hsin-Fu Yeh, Hung-Hsiang Hsu

**Affiliations:** Department of Resources Engineering, National Cheng Kung University, Tainan 701, Taiwan; yohawnhsu@gmail.com

**Keywords:** thermal water, hydrochemistry, Tayukeng, Tatun volcano group

## Abstract

The Tatun Volcano Group (TVG) is located in northern Taiwan and consists of many springs and fumaroles. The Tayukeng (TYK) area is the most active fumarole site in the TVG. In this study, we analyzed the long-term geochemical variations of hydrothermal fluids and proposed a mechanism responsible for the variation in TYK. There are two different aquifers beneath the TYK area: a shallow SO_4_^2^^−^-rich aquifer and a deeper aquifer rich in Cl^−^. TYK thermal water was mainly supplied by the shallow SO_4_^2−^-rich aquifer; therefore, the thermal water showed high SO_4_^2^^−^ concentrations. After 2015, the inflow of deep thermal water increased, causing the Cl^−^ concentrations of the TYK to increase. Notably, the inferred reservoir temperatures based on quartz geothermometry increased; however, the surface temperature of the spring decreased. We inferred that the enthalpy was lost during transportation to the surface. Therefore, the surface temperature of the spring does not increase with an increased inflow of deep hydrothermal fluid. The results can serve as a reference for understanding the complex evolution of the magma-hydrothermal system in the TVG.

## 1. Introduction

In many volcanic areas, chemical monitoring has been conducted to reveal the state of the volcano, in order to reduce the volcanic hazards [1,2,3,4,5,6,7,8]. The hydrothermal system in the volcanic area usually has a direct connection to the magma system [9]. When the gas released from magma interacts with the water, it is preserved as anions in the thermal water. Therefore, indicators such as SO_4_^2^^−^, Cl^−^, pH, and the temperature of the thermal water can provide information about volcanic activity [6]. Thermal water in volcanic areas that undergo deep formation through the condensation of volcanic vapors or dissolution of acidic magmatic volatiles in groundwater is characterized as acid-sulfate-chloride water [10]. Long-term geochemical variation of the thermal waters is useful for understanding the magmatic-hydrothermal system in volcanic areas. The chemical composition of volcanic thermal water is affected by feeding from different aquifers [3,5] or the pressure and temperature variations in shallow hydrothermal systems [7].

The TVG is situated at the northern tip of Taiwan, close to Taipei, the capital city of Taiwan and two nuclear power plants. There is an abundance of hot springs and fumaroles in the TVG [11], most of which are distributed in the area between the Chinshan and Kanchiao faults. Tayukeng (TYK) is the strongest fumarole in the TVG [12]. The dominant species of the TYK fumarole gas is SO_2_, which shows a more oxidizing magmatic condition [13]. The helium isotope results showed that a magma chamber may exist beneath the TVG [14]. According to seismic observations, seismicity is clustered in a narrow vertical conduit beneath TYK, induced by ascending volcanic gases or fluids [15]. Recent studies have conducted geophysical investigations of the TVG, such as precise leveling [16], gravity monitoring [17], satellite images [18] and seismological observations [19] indicating that hydrothermal activity is still significant in the TVG, and the possibility of future volcanic eruption should not be eliminated.

In order to mitigate volcanic hazards, it is important to understand the current state of the volcano. The geophysical observations can provide spatial information of hydrothermal systems and heat sources [7], geochemical investigations can help to understand the processes in the volcanic hydrothermal system [20]. Combine geophysical and geochemical results, we can better understand the volcanic hydrothermal system [2,7]. Numerous studies at the TVG have focused on geophysical monitoring, included seismicity, deformation, and gravity [16,17,19]. The chemical component variations are also useful for evaluating the current state of the magmatic-hydrothermal system. The geochemistry of fumarolic gases between 2003 to 2006 had also been investigated [21]. In this study, we analyzed the geochemical monitoring data of TYK thermal water between 2004 and 2019. We focused on the temperature and magmatic compositions such as Cl^−^ and SO_4_^2^^−^ and compared the results with surface deformation observations to propose the mechanism responsible for the chemical composition variations. Additionally, we discuss the interactions between the deep Cl^−^-rich aquifer and the TYK spring, and the characteristics of the aquifers beneath the TVG.

## 2. Study Area

**Figure 1:**
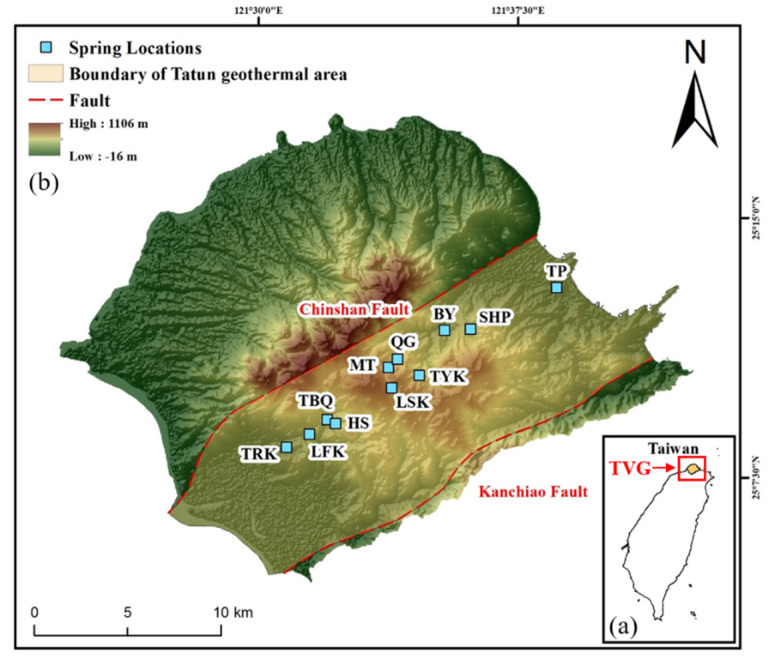
(**a**) Inset map of Taiwan showing the location of Tatun Volcano Group (TVG); (**b**) a map of TVG highlighting geothermal areas (springs, fumaroles).

The TVG is situated at the northern tip of Taiwan. The Tatun Volcano Group contains at least 20 volcanoes covering an area of 400 km^2^ [22]. The Chinshan and Kanchiao faults are the two major faults in the TVG: both strike NW and dip SW. The geothermal area in the TVG is bound by these two major faults. The thermal springs and fumaroles in the TVG are mainly distributed along the Chinshan fault, as shown in Figure 1. TVG is underlain by sedimentary basement rocks, and the sedimentary basement form part of the geothermal reservoir section [23]. The Tatun volcanic rocks consist of lava flows, pyroclastic breccias, surges, tuffs, lahars, and reworked volcaniclastic rocks; the Wuchihshan Formation consists of porous quartz-rich sandstone is the most important unit of sedimentary sequences for the Tatun geothermal system, most of the hot water is stored in this formation [13]. Volcanic activity at the TVG is divided into two stages: the first stage (2.8–2.5 Ma) and the second stage (0.8–0.35 Ma) [24]. According to ^14^C dating, some volcanoes had magmatic eruptions between 23,000 and 13,000 years ago, and possibly a phreatic eruption at Mt. Cising 6000 years ago [25]. No historical eruptions are recorded in the TVG. However, geodetic, geophysical, and geochemical observations indicate that an active magma chamber may still exists beneath TVG [14,16,19,21].

## 3. Methodology

### 3.1. Data Source

The TYK spring geochemical data analyzed in this study were collected from the Tatun Volcanic area spring quality analysis data [26]. The seismic data of the TYK area were obtained from government reports [27].

### 3.2. Method of Data Analysis

Correlation analysis can be used to analyze the water chemistry, investigate the supply resources and the hydrochemical characteristics of geothermal fluids [28,29]. In this study, a correlation coefficient analysis was employed to calculate the statistical relationship between the two variables. The equation of Pearson’s correlation is defined as:

(1) $$r=\frac{\sum_{i=1}^{n}\left(x_{i}-x)(y_{i}-y\right)}{\sqrt{\sum_{i=1}^{n}{\left(x_{i}-x\right)}^{2}\sum_{i=1}^{n}{\left(y_{i}-y\right)}^{2}}}$$

where x and y are the means of x_i_ and y_i_, respectively. The value of the Pearson correlation coefficient, r, ranges between −1 and 1. An r value of 1 indicates a positive association, and towards −1 indicates a negative association between the two variables. A value of 0 indicates that there is no relationship between variables. The Statistical Package for the Social Sciences (SPSS) version 17 was used to obtain Pearson’s correlation.

### 3.3. Geothermometer

Geothermometers are based on the solubility and the equilibrium of the minerals in the reservoir, which are affected by the temperature at different ranges; therefore, they have been widely applied to estimate the reservoir temperature [30,31,32]. The hot water in the TVG is stored in the Wuchihshan Formation, which consists of quartz-rich sandstone [33]. In addition, most of the springs in the TVG reach an equilibrium with quartz [34]. To determine the variation in reservoir temperature, the quartz geothermometer proposed by Fournier and Potter [35] was applied in this study, which is defined as:

(2) $$T=-42.2+{0.28831(\mathrm{SiO}}_{2})\;-\;3.6686\times 10^{-4}{{(\mathrm{SiO}}_{2})}^{2}+3.1665\times 10^{-7}{{(\mathrm{SiO}}_{2})}^{3}+77.034\log\left({\mathrm{SiO}}_{2}\right)$$

where T is the estimated reservoir temperature in degrees Celsius, and SiO_2_ is the silica concentration in mg/L.

### 3.4. Hydrochemical Analysis

The liquid-analysis spreadsheet proposed by Powell and Cumming [36] was used to analyze the hydrochemical characteristics of thermal water in this study. The spreadsheet integrates numerous types of analysis methods, which have been widely used in hydrochemical studies [32,37].

## 4. Results and Discussion

### 4.1. Chemical Variation of TYK Thermal Water

**Figure 2:**
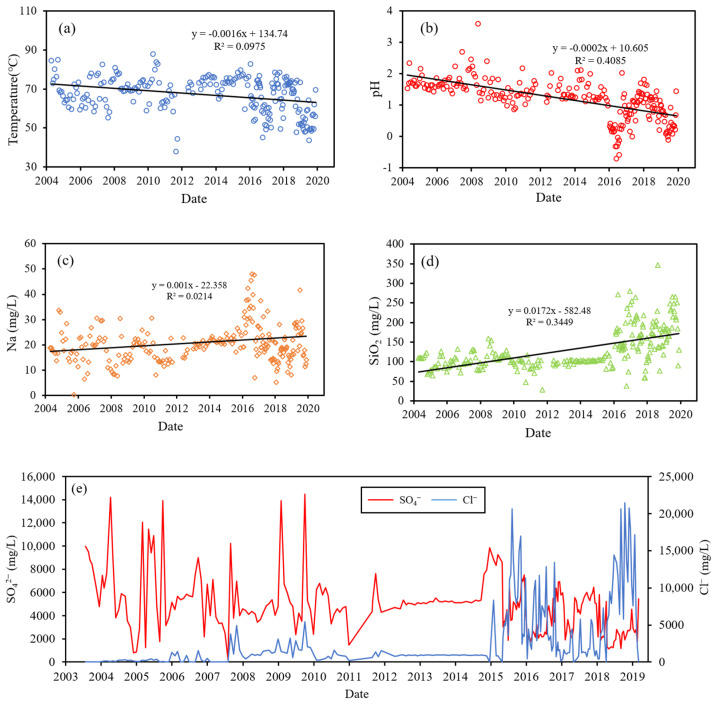
Temporal variation of (**a**) temperature, (**b**) pH, (**c**) Na concentration, (**d**) SiO_2_ concentration, and (**e**) Cl^−^ and SO_4_^2^^−^ concentration of Tayukeng (TYK) thermal water.

The temperature, pH, and main chemical component variation of the TYK thermal water is shown in Figure 2. From 2004 to 2019, the overall temperature range is from 37.8 °C to 88 °C and the pH range is from −0.7 to 3.59, both of which show a declining trend. The concentrations of Na^+^ and SiO_2_ showed an increasing trend. The concentrations of SO_4_^2^^−^ showed significant fluctuations, expect for the period between 2012 and 2015 where the concentrations remained stable. The concentration of Cl^−^ noticeably increased and showed significant variation after 2015.

**Figure 3:**
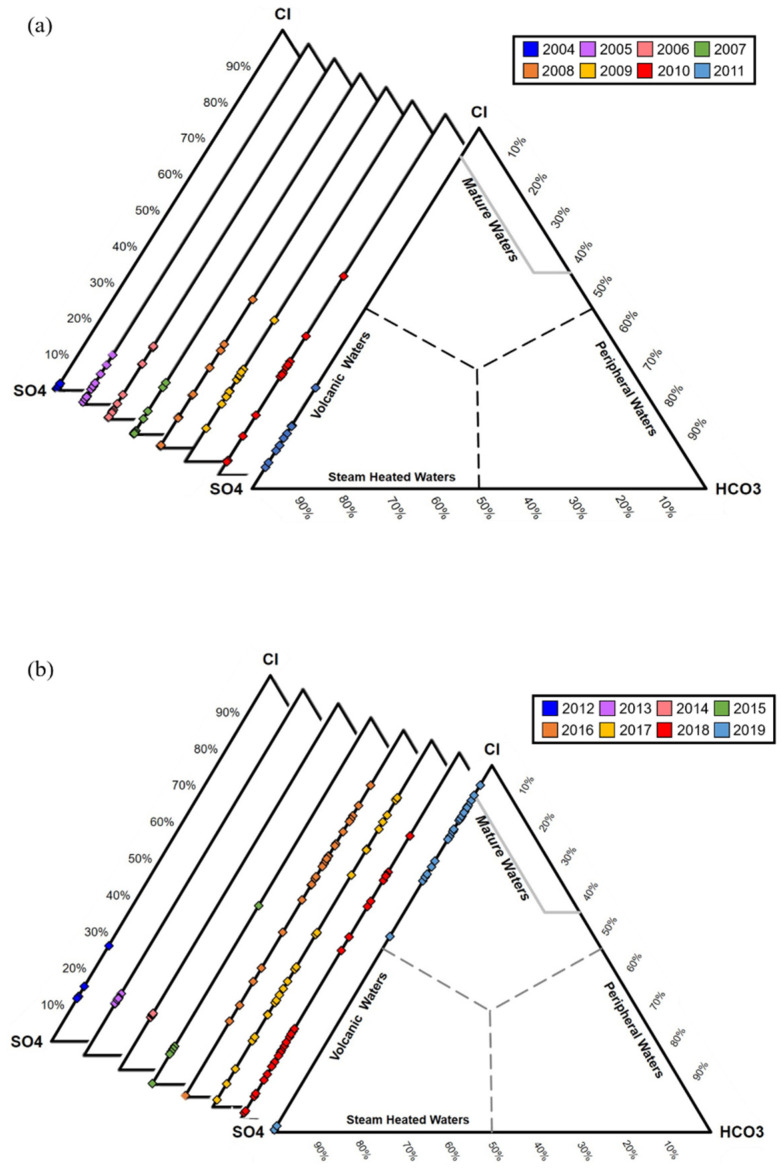
Cl-SO_4_-HCO_3_ ternary diagrams for TYK thermal water: (**a**) 2004–2011; (**b**) 2012–2019.

The proportions of Cl^−^, SO_4_^2^^−^, and HCO_3_ are commonly used to characterize different types of geothermal water, such as volcanic and steam-heated waters, mature NaCl waters, and peripheral waters [38]. The Cl-SO_4_-HCO_3_ ternary plot of the TYK thermal water is shown in Figure 3. The thermal water of TYK is predominantly acid-sulphate between 2004 and 2015. After 2016, the Cl^−^ concentration increased, indicating a supply from different aquifers. Similar temporal changes in chemical composition occur in several volcanic areas, influenced by different aquifers [5,39,40].

### 4.2. Variation of Cl^−^ and SO_4_^2^^−^

In volcanic geothermal systems, the SO_4_^2^^−^ and Cl^−^ content in the thermal water originate from the acidic gas (SO_2_, HCl) released from the magma [4,6,10]. In numerous volcanic areas, SO_4_^2^^−^ and Cl^−^ are useful for understanding the magmatic-hydrothermal system or serve as indicators for volcanic activity [3,7,10].

**Figure 4:**
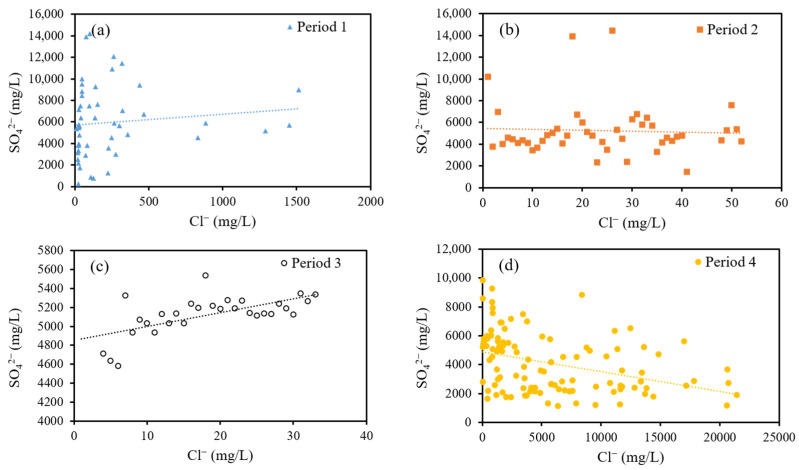
SO_4_^2−^ versus Cl^−^ concentration of TYK thermal water in (**a**) period 1, (**b**) period 2, (**c**) period 3, and (**d**) period 4.

SO_4_^2^^−^ and Cl^−^ are the predominant components in the TYK thermal water, where Cl^−^ originate from formation waters in the Wuchihshan sandstone and SO_4_^2^^−^ originate from magmatic fluid [41]. It can be seen that the Cl^−^ and SO_4_^2^^−^ concentrations seem to have changed according to different trends, as shown in Figure 2. The variation of Cl^−^ (constantly above 1500 mg/L) was less than that of SO_4_^2^^−^ (14,197–243 mg/L) between 2004 and 2008. From 2008 to 2012, the SO_4_^2^^−^ concentrations still showed significant variation, and the variation in Cl^−^ concentrations increased. The Cl^−^ and SO_4_^2^^−^ concentrations were almost constant from 2012 to 2015. After 2015, the Cl^−^ concentrations significantly increased and showed the largest variations; the SO_4_^2^^−^ concentrations varied and showed a declining trend. In this study, we used the correlation coefficient to separate the data into four periods, as shown in Table 1. In period 1 (2004/4–2008/5), Cl^−^ and SO_4_^2^^−^ concentrations were not significantly related. In period 2 (2008/5–2012/9), a positive correlation was found between Cl^−^ and SO_4_^2^^−^, and a correlation coefficient of 0.469 was found to be statistically significant at 0.01. In period 3 (2012/9–2015/6), Cl^−^ and SO_4_^2^^−^ concentrations also showed a positive correlation coefficient of 0.589, but their concentrations remained constant during this period. In period 4 (2015/6–2019/12), a negative correlation was found between Cl^−^ and SO_4_^2^^−^ concentrations; the correlation coefficient of −0.369 was found to be statistically significant at 0.01. Furthermore, the regression line of period 4 is different from that of the other periods, as shown in Figure 4. We infer that the supply aquifer of the TYK was different from the other periods.

**Figure 5:**
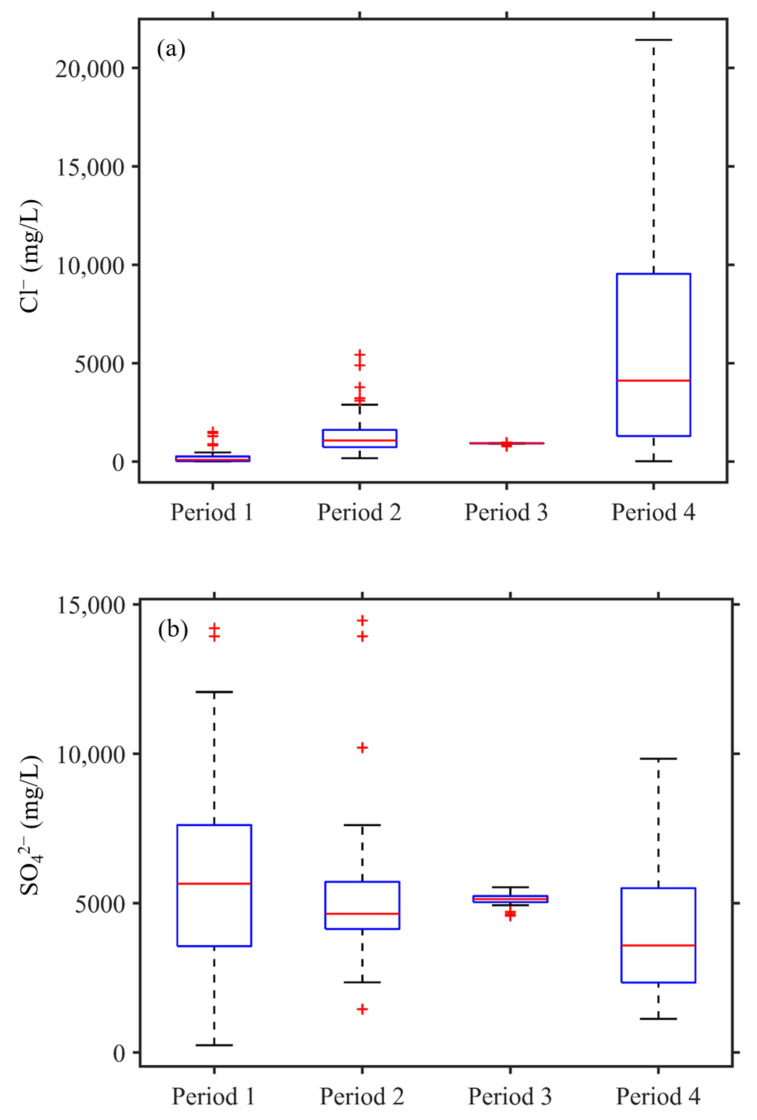
Box plot of (**a**) Cl^−^ and (**b**) SO_4_^2^^−^ in different periods.

The summary statistics and box plots of Cl^−^ and SO_4_^2^^−^ concentrations for each period are shown in Table 2 and Table 3 and Figure 5. The variability of Cl^−^ and SO_4_^2^^−^ was different in each period for the TYK. Sulfates exhibited similar mean concentrations during each period. The range and mean of Cl^−^ concentrations showed significant difference during period 4. In many volcanic geothermal areas, thermal water with high Cl^−^ concentrations represents deep geothermal water [3]. In this study, we focused on the relationship between Cl^−^ concentrations and deep geothermal fluid transportation.

### 4.3. Supply of Deep Cl^−^-Rich Aquifer

In many volcanic areas, temporal variations in Cl^−^ concentration in thermal water can serve as an effective indicator of magmatic fluid migration [2,3,7]. In some cases, seawater could influence the compositions of spring water [42]. Some of the springs near the sea in the northeast of TVG are affected by seawater [43]. However, according to previous studies on the geochemical distribution of the springs and fumarolic gases, the composition variations of the springs in the middle and southwest of the TVG could be contributed by formation water or magmatic fluid [21,41].

**Figure 6:**
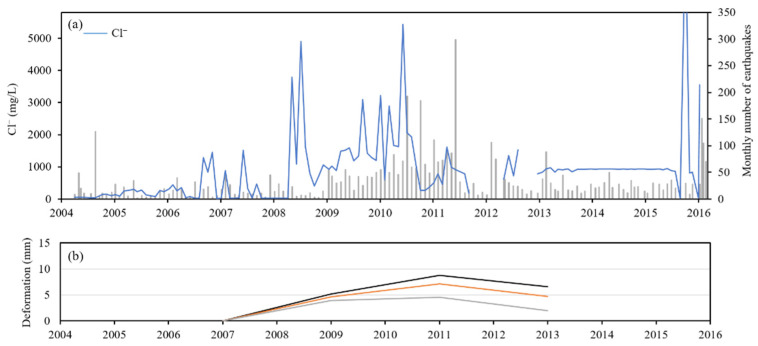
Correlation between the Cl^−^ concentration of TYK waters and (**a**) monthly number of earthquakes. (**b**) Vertical deformations of benchmarks in TYK area according to [16].

Some of the earthquakes in the TVG were associated with volcanism [12,19,44], as there was a clustering seismicity conduct nearby TYK, probably triggered by ascending volcanic gases and fluids [15]. The ascent of fluids may influence the composition of the springs. A correlation was observed between the Cl^−^ concentration of TYK thermal water and the seismic activity, as shown in Figure 6. The Cl^−^ concentration and seismic activities had larger variations in period 2 (2008–2012), and the Cl^−^ concentration remained constant in period 3 (2012–2015), when the seismic activity was relatively quiescent, indicating that some of the earthquakes were related to ascent deep aquifer and had affected the Cl^−^ concentration of TYK thermal water.

In 2004, new fractures opened in TYK area, more magmatic or deep thermal fluid ascended and caused the fumarolic gas composition variations [21]. Meanwhile, the Cl^−^ concentration slightly increased, as shown in Figure 6. Furthermore, the temporal deformations at TYK area are also consistent with the Cl^−^ concentration variations in TYK thermal water. Precise leveling survey had been conducted at TVG during 2006 to 2013 [16]. The variations in Cl^−^ concentrations in the TYK thermal water was consistent with the results from ground deformations, as shown in Figure 6. In period 2 (2008–2012), the variations in Cl^−^ concentrations were larger than those in period 1 and ground uplift was detected. In period 3 (2012–2015), the Cl^−^ concentrations remained constant and slight subsidences were detected. The variations in Cl^−^ concentrations in the TYK thermal water show a good correlation with seismic activity and deformations, indicating the TYK thermal water was influenced by the supply of a deep aquifer. The Cl^−^ concentrations of the TYK thermal water can serve as an indicator of the deep aquifer supply.

The Cl^−^ concentrations in period 4 had the largest variation, and the Cl^−^ mean concentrations increased from 916 mg/L to 5919 mg/L when compared to period 3. Notably, the correlation coefficient of Cl^−^ and SO_4_^2^^−^ was −0.369 in period 4, which was different from the other periods. As described above, we interpret this phenomenon to be related to deep aquifer supply during period 4. In order to understand the characteristics of deep aquifer, we applied correlation analysis on the hydrochemical data of TYK in period 4. The correlation of the chemical and physical parameters is shown in Table 4. Total Dissolved Solids (TDS), Ca^2+^, Na^+^, Al^3+^, and Si are positively correlated with Cl^−^, and negative correlations are observed for Cl^−^ with pH, Fe^2+^, SO_4_^2^^−^, and spring temperature. Based on the results of the correlation analysis, the deep aquifer is rich in Cl^−^, Na^+^, and Si. In addition, there may exist a high temperature shallow SO_4_^2^^−^-rich aquifer, which may be due to the absorption of volcanic vapors. Geothermal drilling data also supports the characteristics of deep aquifer obtained from correlation analysis [33]. In the Tokachidake volcano, a negative correlation was found between Cl^−^ and SO_4_^2^^−^, and the inflow from aquifers changed with different volcanic activity [3].

### 4.4. Application of Silica Geothermometry

**Figure 7:**
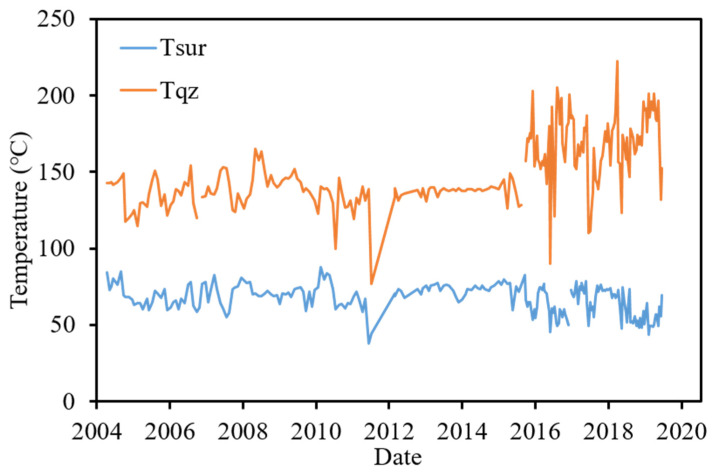
Surface and quartz geothermometer temperature time series of TYK thermal water.

The chemical composition of thermal water is often used to estimate the reservoir temperature [5,30,31,32]. Most of the springs in the TVG do not reach equilibrium with minerals, and cation geothermometry may provide uncertain results [34]. Most of the thermal water is entrapped in the quartz-rich sandstone of the Wuchihshan Formation. In this study, quartz geothermometry (Equation (2)) was used to estimate the reservoir temperature, as shown in Figure 7 and Table 5. There was no clear correlation between the spring and reservoir temperatures. The mean temperature of the spring was 69 °C in periods 1 and 2, increased to 73.75 ℃ in period 3, and decreased to 63.45 °C in period 4. The mean reservoir temperature is approximately 136–138 °C in period 1, 2, and 3, and increased to 165 ℃ in period 4. Notably, the mean reservoir temperature increases with time, but the mean spring temperature decreases in period 4. The estimated reservoir temperature variation could be caused by the deep reservoir temperature increase or the increase in the supply from the deep aquifer. If the reservoir temperature increases, the spring temperature should simultaneously increase. Therefore, the temperature of deep thermal waters may be lost during transport to the surface. The reservoir temperature rise may be caused by an increase in supply from deep aquifer.

## 5. Conclusions

Long-term geochemistry data of thermal fluids is useful for understanding hydrothermal systems in volcanic areas. In this study, we analyzed the variation in the chemical composition of the TYK thermal water between 2004 and 2019. The results showed that at least two thermal aquifers existed beneath the TYK area. The variations of Cl^−^ and SO_4_^2^^−^ in the thermal water were caused by the change in the predominant aquifer. The Cl^−^ concentration was affected by the deep hydrothermal aquifer supply. Since 2004, deep aquifer transport through fractures has caused the Cl^−^ concentration to gradually increase. The Cl^−^ concentration remained stable from 2012 to 2015 because the supply from the deep aquifer decreased. After 2015, the Cl^−^ concentration significantly increased, indicating that the supply from the deep aquifer increased. Based on the long-term trends of thermal water chemical compositions, we inferred that the deep aquifer was rich in Cl^−^ and SiO_2_. With the increased supply of deep aquifer fluid, the TYK spring temperature did not increase simultaneously, and the reservoir temperatures decreased. The temperature of deep thermal waters may be lost during transport to the surface. The results can help understand the variation in the hydrothermal system in the TYK area of the TVG.

## Figures and Tables

**Table 1:** Correlation coefficient between Cl^−^ and SO_4_^2^^−^ of Tayukeng (TYK) thermal water in each period.

	2004/4–2008/5	2008/5–2012/9	2012/9–2015/6	2015/6–2019/12
Correlation coefficient	0.11	0.469	0.589	−0.369
Number of samples	50	46	30	109

**Table 2:** Summary statistics for Cl^−^ in different periods.

Cl^−^	Period 1	Period 2	Period 3	Period 4
Minimum	10.74	171.90	781.37	23.10
Maximum	1512.50	5430.50	977.16	21,427.00
Range	1501.76	5258.60	195.79	21,403.90
IQR	240.65	879.00	12.33	8243.50
Mean	234.21	1406.56	916.86	5919.47
Median	90.79	1076.75	928.05	4118.00
Variance	126,956.50	1265,263.94	1592.47	3.047 × 10^7^
Std	356.20	1124.83	39.90	5520.09

**Table 3:** Summary statistics for SO_4_^2^^−^ in different periods.

SO_4_^2^^−^	Period 1	Period 2	Period 3	Period 4
Minimum	242.35	1452.60	4581.37	1126.00
Maximum	14,197.00	14,457.50	5535.00	9839.00
Range	13,954.65	13,004.90	953.63	8713.00
IQR	4313.28	1601.38	211.25	3188.50
Mean	5937.97	5233.19	5123.39	4064.69
Median	5653.66	4647.37	5138.50	3588.00
Variance	1.086 × 10^7^	5824,309.15	42,138.68	4,228,843.15
Std	3295.28	2413.36	205.27	2056.41

**Table 4:** Correlation analysis of the main ions of TYK thermal fluid in period 4.

	TDS	pH	Ca	Mg	Na	K	Fe	Al	Cl	SO_4_	Si	T
**TDS**	1	−0.71	0.497	−0.51	0.586	0.226	−0.39	0.408	0.828	−0.211	0.306	−0.36
**pH**		1	−0.481	−0.42	−0.513	−0.035	0.152	−0.383	−0.676	0.13	−0.407	0.183
**Ca**			1	0.56	0.193	0.079	−0.249	0.286	0.509	−0.374	0.462	−0.498
**Mg**				1	0.025	0.004	0.104	0.221	0.012	0.045	0.263	−0.142
**Na**					1	0.198	−0.15	0.489	0.487	0.119	0.074	0.05
**K**						1	0.178	0.615	0.181	0.285	0.136	0.243
**Fe**							1	0.001	−0.501	0.425	−0.01	0.539
**Al**								1	0.306	0.277	0.254	0.111
**Cl**									1	−0.369	0.432	−0.539
**SO_4_**										1	−0.184	0.732
**Si**											1	−0.29
**T**												1

**Table 5:** Average surface and quartz geothermometer temperature of TYK in each period.

	Period 1	Period 2	Period 3	Period 4
Tsur(°C)	69.59	68.66	73.75	63.45
Tqz(°C)	135	137	138	165

(Tsur: surface temperature, Tqz: quartz geothermometer temperature).

## Data Availability

Publicly available datasets were analyzed in this study. This data can be found here: https://data.gov.tw/dataset/16794 (accessed on 26 April 2021).
